# Exposure to multiple metallic elements and risk of thyroid tumors: insights from elemental profiling, diet, and molecular characteristics plasma levels of metallic elements

**DOI:** 10.3389/fonc.2026.1840392

**Published:** 2026-06-18

**Authors:** Chao Zhai, Jianyun Sheng, Liang Chen, Long Jia, Chaoyang Wang, Tuo Han, Peina He, Shushen Ji, Mengxin Zhao, Dong Xiao

**Affiliations:** 1 Department of Thyroid and Breast Surgery, 3201 Hospital of Xi’an Jiaotong University Health Science Center, Han-Zhong, Shaanxi, China; 2 Department of General Surgery, Pingdingshan First People’s Hospital, Pingdingshan, Henan, China; 3 School of Medicine, Pingdingshan University, Pingdingshan, Henan, China; 4 Shanghai Biotecan Medical Laboratory Co., Ltd., Shanghai Zhangjiang Institute of Medical Innovation, Shanghai, China

**Keywords:** BRAF V600E mutation, dietary habits, papillary thyroid carcinoma, thyroid function parameters, thyroid neoplasms, trace elements

## Abstract

**Background:**

The global incidence of thyroid tumors has been on a steady increase. Although it is hypothesized that environmental exposures, especially to metallic elements, have an etiological role, their specific associations with the risk of thyroid disease, potential interactions with dietary habits, and connections to molecular characteristics such as the BRAF V600E mutation are still inadequately characterized. The objective of this study was to investigate plasma metal levels, their association with diet, and potential links to molecular features in papillary thyroid carcinoma (PTC).

**Methods:**

This hospital-based case-control study recruited 1, 046 patients with pathologically confirmed thyroid diseases (including papillary thyroid carcinoma and nodular goiter) and 854 healthy controls from Hanzhong, China. The concentrations of 18 metallic elements in plasma/serum were quantified using inductively coupled plasma mass spectrometry (ICP-MS). The BRAF V600E mutation status was determined in a subset of papillary carcinoma cases.

**Results:**

Patients with thyroid disease showed significantly altered plasma elemental profiles compared to healthy controls. Dietary habits had a significant influence on elemental concentrations; for example, the consumption of Youtiao (fried dough) was associated with a value 153% above the upper limit of the normal reference range. Moreover, specific alterations in elemental profiles were significantly correlated with abnormal thyroid hormone levels. Among the PTC cases, the BRAF V600E mutation (identified in 51%) showed a non-significant tendency towards an association with higher systemic levels of mercury and cadmium.

**Conclusions:**

This study reveals distinct alterations in the plasma metallic element profiles of patients with papillary thyroid carcinoma (PTC), which are significantly influenced by dietary habits. Our findings provide new perspectives on the intricate interaction between lifestyle factors and the molecular pathogenesis of thyroid tumors, emphasizing potential directions for prevention and future research.

## Introduction

Thyroid cancer is one of the most rapidly growing malignancies globally and currently ranks among the most prevalent cancers, with an estimated 821, 214 new cases worldwide in 2022 (1). Differentiated thyroid cancer (DTC), especially papillary thyroid carcinoma (PTC), represents vast majority of thyroid malignancies (approximately 85–95%), thus being the most prevalent endocrine tumor (2). Nodular thyroid diseases, such as nodular goiter, exhibit a high prevalence in the general population. A recent meta - analysis estimated that the overall prevalence of thyroid nodules (detected by various methods) is approximately 24.8% (95% CI 21.4–28.6%), and the prevalence is higher in women (approximately 36.5%) than in men (approximately 23.5%) (2). Although advancements in diagnostic techniques and heightened health awareness have led to increased detection rates of thyroid diseases, mounting evidence indicates that environmental and dietary factors also have significant impacts on thyroid tumorigenesis (3–6). In particular, exposure to various metallic elements and trace elements—including toxic heavy metallic elements (e.g. arsenic, cadmium, mercury, plumbum) and essential trace elements (e.g. iodine, selenium, zinc) — has been associated with thyroid dysfunction and potentially to carcinogenesis, through mechanisms such as oxidative stress, endocrine disruption, immune imbalance, and altered cellular proliferation in thyroid diseases (7–16). However, existing studies present inconsistent and sometimes conflicting findings regarding the distribution patterns and biological effects of these elements in benign versus malignant thyroid tissues, which likely reflects differences in geographic region, exposure source, and dietary habits (17–22). Therefore, a comprehensive comparative analysis of element level between thyroid cancer and benign thyroid diseases is still required to elucidate their potential roles in the development and progression of thyroid disorders (23, 24).

Metallic elements are ubiquitous in the environment, and humans are exposed to them through multiple routes, including inhalation, dermal contact, drinking water, and dietary intake (18, 19, 24). While certain trace elements are essential for normal metabolic and endocrine functions, potentially toxic elements (PTEs), including heavy metallic elements, may disturb thyroid homeostasis even at low exposure levels over time (21, 24). Epidemiological studies have increasingly implicated metal exposures in thyroid dysfunction and disease risk. For example, exposure to metal mixtures, such as arsenic, cadmium, and plumbum, has been associated with altered thyroid hormone levels (T3, T4, TSH), suggesting that environmental-level exposures may disrupt thyroid endocrine regulation (25–28). Human case–control studies further reported that serum or tissue levels of metallic elements including chromium, manganese, nickel, cadmium, and mercury differ between individuals with thyroid tumors or goiter and healthy controls, implying that elemental imbalances may influence thyroid disease susceptibility (29–34). Despite accumulating evidence, results remain inconsistent across studies, likely due to differences in exposure sources, geographic background, metal species examined, co-exposures, and analytical methods, highlighting the need for systematic comparative investigations of metal profiles in benign and malignant thyroid disorders (24, 29, 33).

Furthermore, existing research has primarily focused on the influence of metallic elements on thyroid hormones, with relatively few studies examining their associations with the risk of thyroid tumor and goiter. Most of the limited studies reported that certain metallic elements may act as thyroid endocrine disruptors, potentially affecting thyroid function and disease susceptibility (35–41). Clearly, more epidemiological investigations are needed to comprehensively evaluate the relationships between mineral element exposure and the occurrence of thyroid tumors and goiter. Although urinary mineral concentrations have been widely used as indicators of internal exposure in humans, reflecting dietary intake and long-term accumulation of elements such as iodine (31, 34), direct measurement of metallic elements in plasma may provide a more immediate and reliable assessment of systemic exposure. Therefore, in the present study, we focused on determining trace and heavy metal levels in plasma samples of participants, aiming to validate and extend the understanding of how metallic elements relate to thyroid tumor and goiter risk.

To further investigate the potential relevance of mineral element exposure to thyroid disease in adults, this study focused on papillary thyroid carcinoma and nodular goiter. Plasma concentrations of eighteen elements - vanadium (V), chromium (Cr), manganese (Mn), cobalt (Co), nickel (Ni), copper (Cu), zinc (Zn), gallium (Ga), arsenic (As), selenium (Se), strontium (Sr), cadmium (Cd), tin (Sn), antimony (Sb), barium (Ba), mercury (Hg), thallium (Tl), and plumbum (Pb) - were determined using inductively coupled plasma mass spectrometry (ICP-MS). By systematically analyzing plasma levels of these elements in relation to both benign and malignant thyroid disorders, this study provides a novel perspective on the role of metallic elements in thyroid pathophysiology. To the best of our knowledge, this is the first study to comprehensively investigate the associations between plasma mineral element profiles and the risk of thyroid tumors and goiter, offering important insights into potential biomarkers and environmental risk factors for thyroid disease.

## Materials and methods

### Study population

This study employed a hospital-based case-series design. Participants were selected from Hanzhong 3201 Hospital, China. The study included patients diagnosed with thyroid tumors (papillary thyroid carcinoma) and benign nodular thyroid disease (colloid or follicular nodular disease). Inclusion criteria were: age 18–75 years, confirmed thyroid disease *via* histopathological biopsy (according to the National Comprehensive Cancer Network (NCCN) guidelines), and residence in the Hanzhong area for over 6 months.

**Figure 1:**
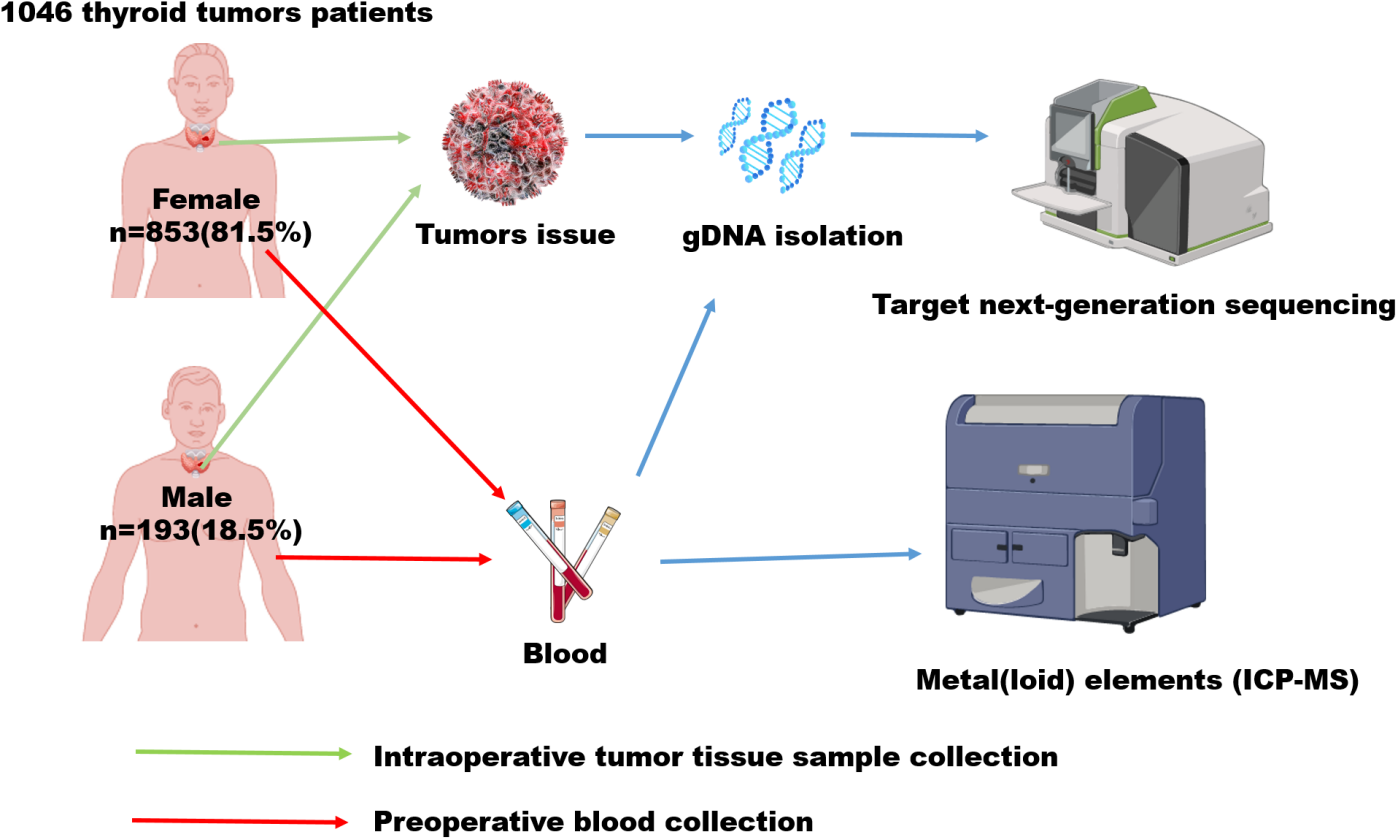
Workflow of study design.

Exclusion criteria comprised: severe heart, liver, or kidney diseases; terminal cancer patients; those who had undergone chemotherapy or radiotherapy; pregnant women; and individuals taking iodine supplements, thyroid hormone medications, contraceptives, or estrogens recently. Demographic characteristics, including age, gender, height, weight, occupation, smoking history, alcohol consumption, dietary habits (e.g., preference for oily or salty foods), and family history of cancer, were collected through structured questionnaires. Body mass index (BMI) was calculated as weight (kg) divided by height squared (m²). Smoking was defined as consuming ≥1 cigarette per day for over 6 months, and alcohol drinking as consuming alcoholic beverages ≥1 time per week for over 6 months. A total of 1900 participants (1046 patients with thyroid disease and 854 healthy controls) were initially enrolled. After applying inclusion/exclusion criteria and data quality control, 760 participants (comprising both cases and controls) had complete data for the primary elemental and thyroid function analysis and constituted the final analytical cohort. The study design was shown in **Figure 1**.

### Sample collection

Serum samples, surgical tissue specimens, fine-needle aspiration samples, and whole blood samples were collected from each participant. Samples were obtained under fasting conditions (≥8 hours) preoperatively, immediately transported to the laboratory, aliquoted into centrifuge tubes, and stored at −80 °C until analysis. Epidemiological data were gathered by trained investigators using standardized questionnaires to ensure consistency.

Surgical tissues are used for pathological diagnosis and immune histochemical analysis. Fine-needle aspiration samples are employed for cytological evaluation. Whole blood and serum are utilized for measuring thyroid function indicators, trace elements, and molecular biomarkers.

### Metal(loid) element detection

Plasma concentrations of trace elements (vanadium, chromium, manganese, cobalt, nickel, copper, zinc, gallium, arsenic, selenium, strontium, cadmium, stannum, stibium, barium, mercury, thallium, and plumbum) were determined using inductively coupled plasma mass spectrometry (Agilent 7800 ICP−MS, Agilent Technologies, USA). Plasma samples were subjected to microwave−assisted acid digestion in 15 mL polypropylene centrifuge tubes using a mixture of HNO_3_ and H_2_O_2_ prior to analysis. For ICP−MS measurement, digested samples were further pretreated via a 10−fold dilution, whereby 0.5 mL of the digestate was added to a diluent containing 0.5% (v/v) nitric acid and 0.1% (v/v) Triton X−100. Instrumental analysis was conducted at ambient temperature. The ICP−MS operational parameters were optimized as follows: cooling gas flow, 12.5 L/min; auxiliary gas flow, 0.7 L/min; nebulizer gas flow, 0.92 L/min; peristaltic pump speed, 20 r/min; spray chamber temperature, 3 °C; CCT gas flow (8% H_2_ in He), 3.75 mL/min; nickel sampler cone aperture, 1.1 mm. Quantification was performed using external calibration with a series of commercial multi−element standard solutions. Quality control included the analysis of method blanks, duplicate samples, and certified reference materials (Seronorm™ Human Serum) in each analytical batch. Method detection limits (MDLs), calculated as three times the standard deviation of the method blanks. The reliability of the analytical procedure was confirmed by recovery rates of all quality control samples within ±20% of the certified values and correlation coefficients (r) of all calibration curves exceeding 0.999.

### Measurement of thyroid functions

Thyroid Function Indicators: Serum levels of thyroid-stimulating hormone (TSH), triiodothyronine (T3), thyroxine (T4), free T3 (FT3), free T4 (FT4), thyroid peroxidase antibody (TPOAb), and thyroglobulin antibody (TGAb) were measured using an automated chemiluminescence immunoassay analyzer (Maglumi 2000). Reference ranges adhered to NCCN guidelines: T3 (0.69–2.15 ng/L), T4 (52–127 ng/L), FT3 (2–4.2 pg/L), FT4 (8.96–17.2 pg/L), TSH (0.3–4.5 μIU/mL), TPOAb (0–30 IU/mL), TGAb (0–95 IU/mL).

### DNA extraction and quality control

gDNA from fresh frozen tissues was extracted byQIAamp DNA Mini Kit, gDNA from blood by QIAamp DNA Blood Mini Kit, gDNA from FFPE tissues by GeneRead DNA FFPE Kit (Qiagen, Hilden, Germany). Quantity and purity of gDNA were assessed by Qubit 3.0 Fluorometer (Invitrogen, Carlsbad, CA, USA) and NanoDrop ND-1000 (Thermo Scientific, Wilmington, DE, USA). Fragmentation status were evaluated by the Agilent 2200 TapeStation system using the Genomic DNA ScreenTape assay (Agilent Technologies, Santa Clara, CA, USA) able to produce a DNA Integrity Number (DIN). An additional quality control (QC) step to assess fresh frozen tissue DNA integrity was performed using a multiplex PCR approach. Briefly, 30 ng of gDNA were amplified using three different- primers for the GAPDH gene (200–400 base pair), and PCR products was measured using an Agilent 2100 Bioanalyzer (Agilent Technologies, Santa Clara, CA). Then, to estimate fresh frozen tissues gDNA fragmentation, we evaluated average yield ratio (AYR) values, calculated by yield ratio of each amplicon compared with a reference DNA (Promega Madison, WI). WES library preparation and hybridization capture A total of 30 ng of each gDNA sample based on Qubit quantification were mechanically fragmented on an E220 focused ultrasonicator Covaris (Covaris, Woburn, MA). Sheared gDNA (200 ng) was used to perform end repair, and A-tailing and adapter ligation with either Agilent SureSelect XT (Agilent Technologies, Santa Clara, CA) or KAPA library preparation kits (Kapa Biosystems Inc., Wilmington, MA) was performed according to the manufacturer’s instructions. Subsequently, libraries were captured using Agilent SureSelect Human All Exon v.6 (Agilent Technologies, Santa Clara, CA) probes and amplified.

### Illumina sequencing

After QC and quantification by Agilent 2100 Bioanalyzer (Agilent Technologies, Santa Clara, CA) and Qubit 3.0 Fluorometer (Invitrogen, Carlsbad, CA, USA), libraries were sequenced on an Illumina Next 500 platform (IlluminaInc, San Diego, CA, USA) High Output mode, 2×75 cycles.

### Next-generation gene-panel sequencing

DNA samples were analyzed for target-capture sequencing with the SureSelect Target Enrichment Kit on an Illumina HiSeq 2500 platform. The average coverage of the targeted region was 600×, and 95% of the target was covered at > 50×. Sequencing reads were aligned to the human genome (NCBI build 37) with the BWA algorithm on default settings. Finally, 801 cases passed internal quality control and quality matrix and were included in further analyses. Mutations in TERT promoter (C228T, C250T), BRAF V600E, KRAS, and NRAS were analyzed *via* polymerase chain reaction (PCR) or sequencing methods to assess molecular characteristics of tumors.

### Quality control

Rigorous quality assurance protocols were implemented throughout the study. Investigators responsible for questionnaire administration received uniform training to standardize data collection. During sample analysis, each batch included blank controls, duplicate samples, and certified reference materials. The acceptance criteria stipulated that the measured values of quality control samples should be within ±20% of the certified values. The correlation coefficients (r) of the standard curves exceeded 0.999. For data verification, a random 30% of questionnaires were back - checked to ensure accuracy.

### Statistical analysis

Categorical variables were presented as frequencies (percentages), while continuous variables were presented as mean ± standard deviation or median (interquartile range). For group comparisons, Mann–Whitney U tests, chi - square tests, or Wilcoxon rank - sum tests were employed, depending on the data distribution. In the univariate and multivariate models, logistic regression analyses were conducted to estimate the associations between trace elements and the risk of thyroid disease, which were presented as odds ratios (ORs) with 95% confidence intervals (CIs). The models were adjusted for age, gender, body mass index (BMI), smoking status, alcohol consumption, and other covariates. Element concentrations were classified into quartiles based on the distributions of the control group, and trend tests were carried out using median values as continuous variables. Regarding the associations with thyroid function, multiple linear regression was used to assess the relationships between log - transformed trace element concentrations and thyroid function indicators. Percentage changes were calculated as (exp(β)−1)×100%, where β represents the regression coefficient. In the additional analyses, Spearman’s rank correlation was used to evaluate the inter - element relationships; principal component analysis (PCA) was applied to identify the dominant factors contributing to the disease risk; restricted cubic splines (RCS) were used to model non - linear associations (with knots at the 5th, 27.5th, 50th, 72.5th, and 95th percentiles). The analyses were performed using SPSS 21.0 and R 3.6.2, and two - sided p - values ≤0.05 were regarded as statistically significant.

### Sensitivity analysis

Interaction terms assessed effect modifications between significant elements. Stratified analyses (e.g., by gender) evaluated robustness, and variance inflation factors (VIF) <10 confirmed absence of multicollinearity.

## Results

### Baseline characteristics of the study population

Based on the study design and methods outlined in the Introduction and Materials and Methods sections, this analysis presents the baseline characteristics of the study participants, including cases with thyroid diseases (diagnosed from 2020 to 2024) and controls, as summarized in **Table 1**. The study population comprised 854 controls and 1, 046 cases (108 cases diagnosed in 2020, 278 in 2021, 259 in 2022, 346 in 2023, and 55 in 2024), all recruited from Hanzhong 3201 Hospital in China, adhering to strict inclusion and exclusion criteria. Demographic, lifestyle, and clinical variables were collected through structured questionnaires and standardized measurements, with statistical comparisons performed using Mann-Whitney U-tests, Student’s t-tests, and Chi-square tests, as appropriate for data distribution. The control group had a mean age of 46.8 ± 12.6 years, while the case groups exhibited variations in age across years: 51.5 ± 39.2 years in 2020, 46.5 ± 10.6 years in 2021, 45.2 ± 11.0 years in 2022, 46.9 ± 10.6 years in 2023, and 45.1 ± 11.9 years in 2024. Thyroid tumors demonstrated a female-predominant distribution across all subgroups (**Table 1**), with the number of female patients significantly exceeding that of males in each category. A total of 1324 female and 563 male patients were included in the analysis, consistently highlighting the higher prevalence among females.

**Table 1:** Baseline characteristics of study participants, stratified by control and case groups (2020–2024).

Characteristics	Controls (n=854)	2020 cases (n=108)	2021 cases (n=278)	2022 cases (n=259)	2023 cases (n=346)	2024 cases (n=55)
Age (years, Mean ± standard deviation)	46.8 ± 12.6	51.5 ± 39.2	46.5 ± 10.6	45.2 ± 11.0	46.9 ± 10.6	45.1 ± 11.9
Male, n (%)	39.5%	16.2%	16.9%	14.6%	24.0%	12.7%
Female, n (%)	60.5%	83.8%	83.1%	85.4%	76.0%	87.3%
BMI (kg/m2, Mean ± standard deviation)	24.5 ± 3.6	23.3 ± 3.5	23.0 ± 3.4	21.1 ± 3.2	23.7 ± 3.4	24.0 ± 3.9
Smoking, n (%)	15.5%	0.0%	0.0%	0.0%	4.0%	0.0%
Alcohol consumption, n (%)	22.9%	0.9%	0.0%	0.0%	0.0%	0.0%
Type of thyroid disease, n (%)						
Papillary thyroid carcinoma	0%	95.4%	99.3%	100%	92.5%	98.2%
Benign nodular goiter	0%	4.6%	0.7%	0.0%	7.5%	1.8%
Dietary habit						
Diet light	89.3%	15.6%	41.7%	100.0%	37.0%	36.4%
Diet high grease	1.1%	40.4%	40.4%	0.0%	38.2%	34.5%
Diet high protein	77.6%	22.9%	17.9%	0.0%	6.6%	9.1%
Diet oversalt	15.6%	22.9%	6.0%	0.0%	23.4%	25.5%
Diet oversweet	14.7%	22.0%	4.6%	0.0%	6.6%	9.1%
Diet preserved egg	1.1%	12.8%	6.0%	0.0%	0.0%	0.0%
Diet Youtiao	1.1%	12.8%	4.0%	0.0%	11.6%	14.5%
Diet Jellyfish	1.5%	0.9%	4.6%	0.0%	0.0%	0.0%

Mann-Whitney U-tests, Student’s t-tests and Chi-square tests were used to estimate differences of variables between the case and control group according to their distributions.

Although age differences were observed, such as the 2020 cases being older on average, the overall age distribution across groups was relatively comparable, with no consistent trend over time. Gender distribution showed a notable disparity: controls had 39.5% males and 60.5% females, whereas cases were predominantly female, with percentages ranging from 83.8% to 87.3% across years, compared to 12.7% to 16.9% males. This aligns with the higher prevalence of thyroid diseases in females. Body mass index (BMI) was similar between controls (24.5 ± 3.6 kg/m²) and most case groups, though the 2022 cases had a lower mean BMI (21.1 ± 3.2 kg/m²), suggesting potential variations in metabolic factors. Lifestyle factors revealed significant differences. Smoking prevalence was 15.5% in controls but negligible in cases (0.0% in most years, except 4.0% in 2023), indicating that smoking might be less common among thyroid disease patients. Similarly, alcohol consumption was reported by 22.9% of controls but was less frequent in cases (0.0% to 0.9%).Regarding thyroid disease types, papillary thyroid carcinoma constituted the majority of cases in this study cohort (92.5% to 100%), while nodular goiter, a benign condition, accounted for a smaller proportion (0.0% to 7.5%).

Dietary habits exhibited substantial variations. Controls reported high rates of light diet (89.3%) and high-protein diet (77.6%), whereas cases showed diverse patterns: for instance, the 2022 cases had 100% light diet but no high-grease or high-protein intake, while other years had higher proportions of high-grease (up to 40.4%) and oversalt diets (up to 25.5%). Preferences for preserved foods, Youtiao, and jellyfish were generally low but more common in some case groups, such as 12.8% for preserved egg and Youtiao in 2020 cases. These findings suggest that dietary factors may influence thyroid disease risk, as postulated in the Introduction, though the patterns are heterogeneous across years.

Overall, the results demonstrate distinct demographic and lifestyle profiles between controls and thyroid disease cases, providing a foundation for further analysis of metal element associations, as detailed in the subsequent methods. The comparisons underscore the importance of adjusting for covariates like age, gender, and BMI in multivariate models to isolate the effects of trace elements on thyroid disease risk.

### Correlations between plasma trace elements and dietary habits revealed by heatmap and comparative analysis

**Figure 2:**
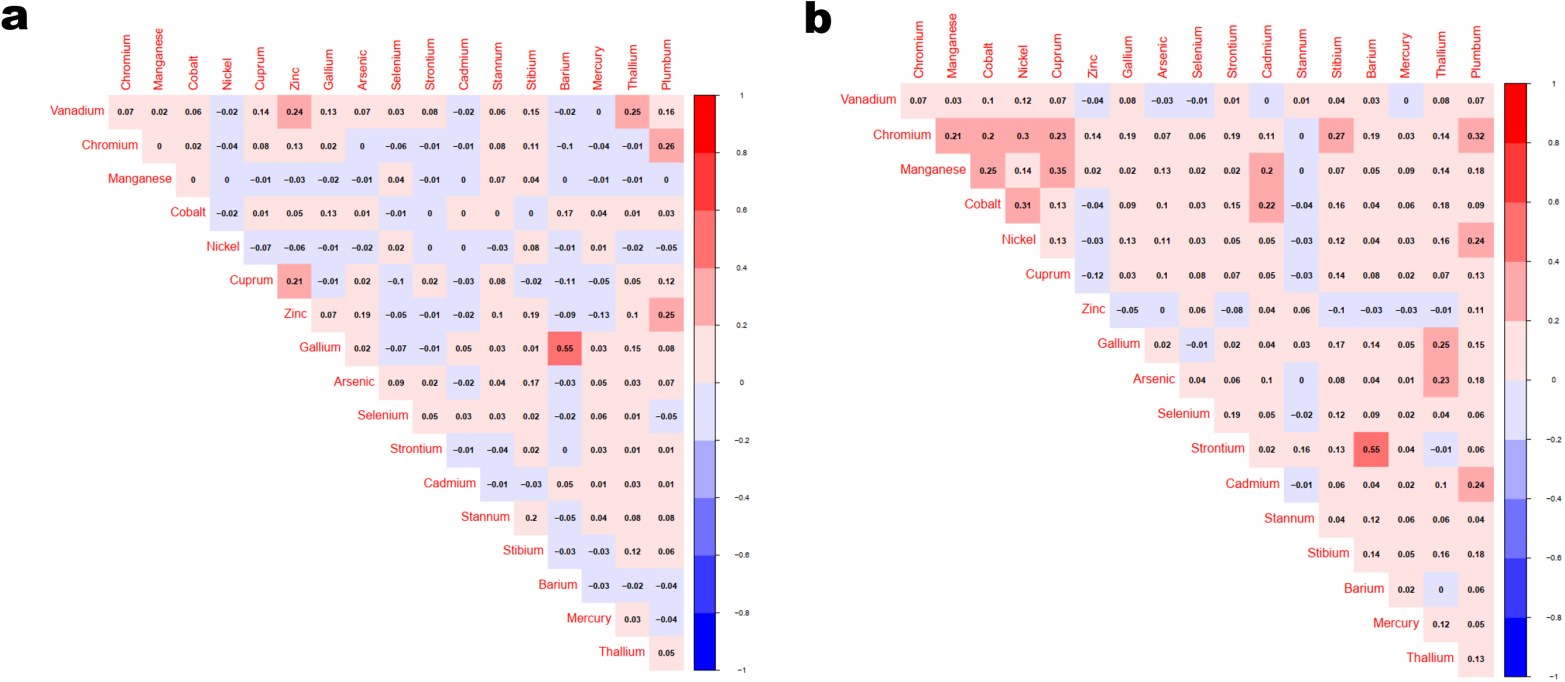
Correlation heatmap of plasma trace metal levels (vanadium, chromium, manganese, cobalt, nickel, cuprum, zinc, gallium, arsenic, selenium, strontium, cadmium, stannum, stibium, barium, mercury, thallium, plumbum) in and 854 healthy individuals as controls **(A)** and 1046 thyroid cancer patients **(B)**.

Pairwise Spearman correlation matrices of 18 trace elements were computed and compared between 854 healthy controls (**Figure 2A**) and 1046 thyroid tumor patients (**Figure 2B**). Visualization with identically scaled color gradients (-1.0 to +1.0) revealed a highly consistent inter-element association network across both cohorts. The overall correlation structure was highly conserved between cohorts, evidenced by a preserved, strong positive cluster among transition metallic elements (V, Cr, Mn, Co). This suggests that core elemental interactions remain stable despite disease status. A notable shift was observed in the associations of Barium (Ba). In healthy controls (2a), Ba showed a positive correlation (r=0.55) with Gallium (Ga). In contrast, within the tumor patient cohort (2b), Ba exhibited a correlation of identical strength (r=0.55) with Strontium (Sr) instead. This substitution in Ba’s primary positive association—from Ga to Sr—may indicate a disease-specific alteration in elemental metabolism or exposure profile, potentially linked to the pathological environment of the tumor. The overall consistency of the correlation matrices implies that fundamental homeostatic networks are largely maintained. However, specific, moderate-level associations like that of Ba may serve as sensitive markers reflecting the unique physiological state or environmental exposures associated with thyroid tumors.

### Plasma trace element profiles and associations with dietary habits

The plasma concentrations of eighteen trace elements measured by ICP-MS are detailed in **Table 2**, comparing healthy controls to patients with thyroid disease and further stratifying patients by dietary habits. Overall, the mean plasma levels of several essential and non-essential elements exhibited notable alterations in the patient group compared to the healthy controls. For instance, patients demonstrated lower mean concentrations of vanadium (0.25 vs. 0.37 μg/L), chromium (2.39 vs. 2.87 μg/L), manganese (13.15 vs. 32.2 μg/L), nickel (0.88 vs. 1.76 μg/L), and selenium (210.09 vs. 250.23 μg/L). Conversely, higher mean levels were observed for zinc (9.38 vs. 7.06 μg/L) and barium (58.51 vs. 48.82 μg/L) in patients. Elements such as mercury showed minimal difference between the groups (3.11 vs. 3.14 μg/L).

**Table 2:** Plasma trace metal and associations with dietary habits.

Elements	Mean value of healthy (μg/L)	Mean value of patients (μg/L)	Mean value of different diet habits in patients							
			Salty	Greasy	Insipid	Sweets	Preserved egg	Youtiao	Jellyfish	High protein
Vanadium	0.37	0.25	0.24	0.23	0.25	0.15	0.21	0.28	0.28	0.25
Chromium	2.87	2.39	2.90	2.51	2.14	2.52	2.05	2.79	2.79	2.41
Manganese	32.2	13.15	13.04	12.92	12.95	13.23	14.60	12.66	12.66	13.47
Cobalt	0.24	0.19	0.21	0.19	0.16	0.21	0.15	0.16	0.16	0.20
Nickel	1.76	0.88	0.91	0.82	0.86	0.72	0.39	0.92	0.92	0.89
Cuprum	880.20	774.23	779.71	776.55	752.04	761.70	823.31	756.58	756.58	804.45
Zinc	7.06	9.38	7.71	7.73	9.70	6.83	6.36	7.67	7.67	6.77
Gallium	0.26	0.25	0.29	0.29	0.22	0.29	0.23	0.31	0.31	0.30
Arsenic	2.71	2.46	2.30	2.19	2.44	2.43	1.72	2.37	2.37	2.10
Selenium	250.23	210.09	202.75	201.51	221.27	194.92	141.17	206.63	206.63	199.36
Strontium	39.25	29.57	33.93	31.88	27.74	28.57	23.02	33.88	33.88	30.31
Cadmium	1.23	1.12	1.15	1.22	1.04	1.15	1.30	1.02	1.02	1.10
Stannum	0.56	0.379	0.39	0.30	0.49	0.20	0.05	0.39	0.39	0.24
Stibium	0.54	0.183	0.27	0.22	0.16	0.20	0.02	0.22	0.22	0.12
Barium	48.82	58.51	78.83	68.47	49.83	56.37	40.81	68.77	68.77	64.29
Mercury	3.14	3.11	3.43	2.80	3.40	6.79	0.14	7.13	7.13	2.32
Thallium	0.10	0.08	0.07	0.10	0.07	0.06	0.04	0.07	0.07	0.06
Plumbum	13.00	13.91	15.21	13.69	13.29	14.68	14.59	14.06	14.06	14.39

Stratification analysis of the patient cohort by dietary habit revealed significant variations in elemental profiles, providing empirical support for the inclusion of dietary patterns as covariates or effect modifiers in risk models. Specifically, patients with a preference for salty or greasy diets exhibited higher plasma concentrations of certain elements, such as barium (78.83 and 68.47 μg/L, respectively), compared to those favoring a light diet (49.83 μg/L). Notably, elevated mercury levels were observed in patients who consumed sweets (6.79 μg/L), with an even higher concentration detected in those who frequently consumed Youtiao (deep-fried dough sticks; 7.13 μg/L). These observations align with known dietary sources of elemental exposure: Youtiao may be a potential source of mercury, jellyfish can accumulate toxic elements such as cadmium, and preserved eggs have historically been associated with lead exposure, despite modern efforts to mitigate this risk. Correspondingly, patients who regularly consumed preserved eggs showed markedly lower circulating levels of several essential elements, including selenium (141.17 μg/L) and antimony (0.02 μg/L). Furthermore, the subgroup adhering to a high-protein diet presented a distinct elemental profile, characterized by lower zinc levels (6.77 μg/L) relative to the overall patient mean. These findings indicate that dietary habits significantly influence the internal exposure and homeostasis of trace elements, thereby potentially modulating their role in the pathogenesis of thyroid disease. The heterogeneity in elemental distribution across dietary subgroups underscores the importance of statistically controlling for dietary patterns as key confounders or effect modifiers when evaluating the association between trace element exposure and thyroid disease risk. Accordingly, in our subsequent risk models (e.g., logistic regression), the aforementioned dietary variables have been included as covariates to derive more robust estimates of the independent associations between elements and disease outcomes.

### Dietary patterns significantly alter element concentrations

**Figure 3:**
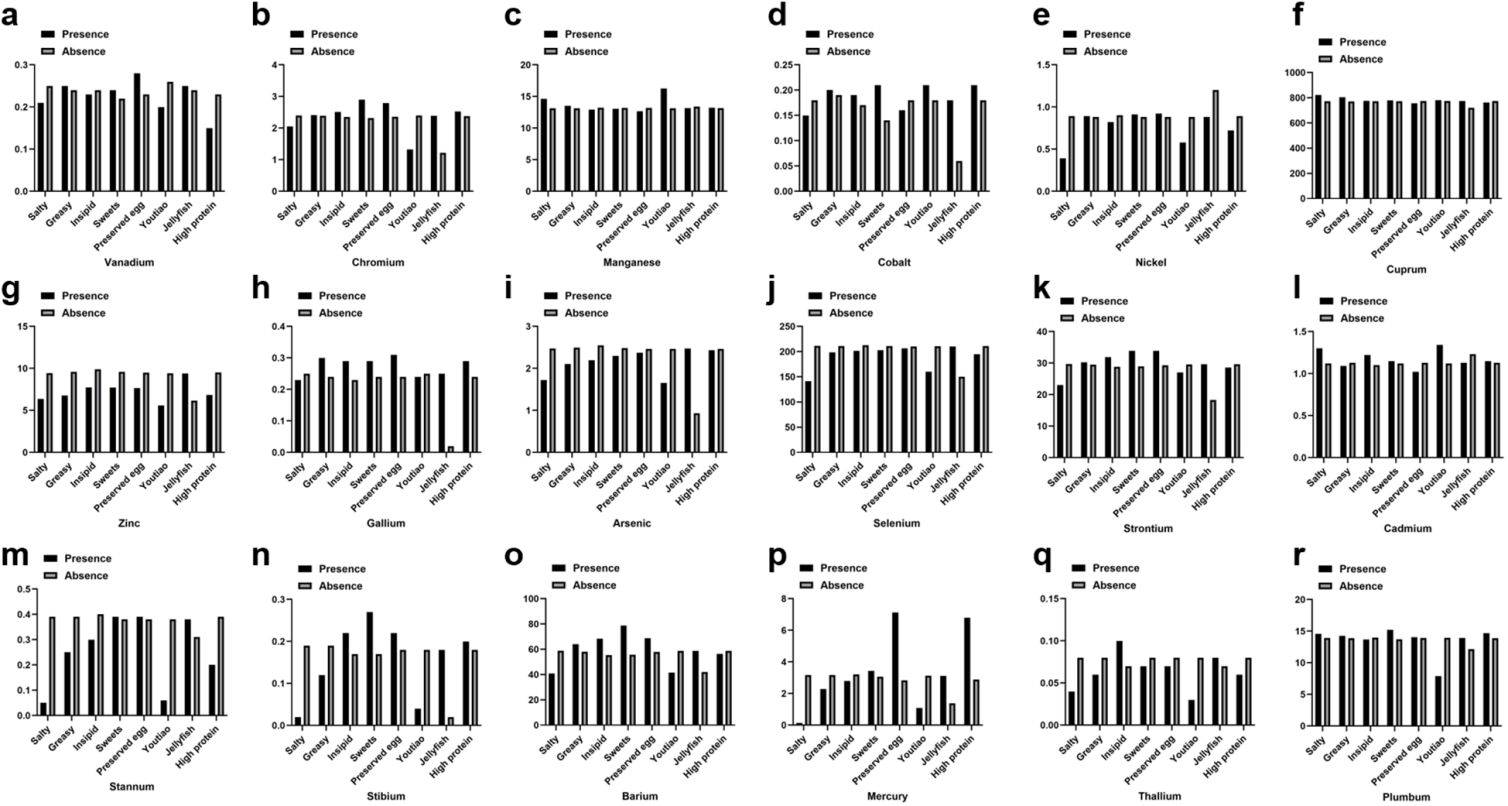
Comparison of element concentrations vanadium **(A)**, chromium **(B)**, manganese **(C)**, cobalt **(D)**, nickel **(E)**, cuprum **(F)**, zinc **(G)**, gallium **(H)**, arsenic **(I)**, selenium **(J)**, strontium **(K)**, cadmium **(L)**, stannum **(M)**, stibium **(N)**, barium **(O)**, mercury **(P)**, thallium **(Q)**, plumbum **(R)**, across various dietary patterns. The bar chart illustrates the levels of different elements under conditions of dietary component “Presence” and “Absence”.

As shown inn **Figure 3**, we systematic analysis of 18 trace elements across seven dietary habit categories reveals distinct patterns in concentration levels when comparing “Presence” versus “Absence” conditions. The most significant and consistent finding across the entire dataset is the exceptionally strong association for Plumbum (**Figure 3R**). The Presence bars for Plumbum are uniformly and markedly higher across all seven food/diet items compared to their Absence counterparts. This indicates a potent linkage between reported consumption of these items and elevated Plumbum levels, suggesting these dietary factors may be notable sources or indicators of Plumbum exposure. Beyond Plumbum, several other elements demonstrate notable, though variable, disparities. Arsenic (**Figure 3H**)​ shows a clear pattern where Presence bars are generally higher than Absence bars for most categories, particularly for salty, creasy, and preserved egg. Similarly, Vanadium (**Figure 3A**)​ and Cadmium (**Figure 3L**)​ exhibit moderate but observable elevations in the “Presence” condition across multiple diet items. This pattern suggests that certain common dietary components may contribute to or correlate with increased bodily concentrations of these elements. In contrast, a group of elements including Chromium (**Figure 3B**), Cobalt (**Figure 3D**), Nickel (**Figure 3E**), and Zinc (**Figure 3G**)​ display relatively minimal differences between Presence and Absence across the majority of dietary categories. The bar heights for both conditions are often closely aligned, indicating that the specified dietary habits may have a less pronounced or more consistent influence on the bodily levels of these essential trace metallic elements. The elements stannum (**Figure 3M**)​and stibium (**Figure 3N**)​present mixed patterns, with significant Presence/Absence differences for only a select few diet items (e.g., sweets for stannum). The data visualizes a spectrum of associations between self-reported dietary habits and elemental concentrations. Plumbum stands out with an alarmingly strong and broad correlation. For elements like arsenic and cadmium, the associations are significant but diet-specific. For another set of elements (e.g., Cr, Co), the dietary factors studied here show limited discriminatory power. These findings highlight specific dietary clusters (particularly those including salty, creasy, and preserved egg) that may warrant further investigation as potential sources of exposure to non-essential trace elements like plumbum and arsenic, while showing lesser influence on certain essential metallic elements. The clear Presence/Absence contrasts serve as strong indicators for generating hypotheses regarding dietary exposure pathways. To conduct a more detailed analysis of the impact of specific diets on trace elements in the blood, we conducted a statistical analysis, as shown in **Table 3**, the analysis of three specific foods (Youtiao, Preserved egg, Jellyfish) corroborates the significant dietary influence on trace element levels. Most strikingly, mercury in Youtiao showed a 153.21% increase compared to non-diet, while preserved egg consumption was associated with markedly lower levels of several elements, particularly mercury (-95.45%), stannum (-87.20%), and stibium (-86.64%). Conversely, Jellyfish intake was linked to a 24.01% higher manganese level. The manganese concentrations observed in this study, particularly in the control group, are higher than some previously reported ranges in general populations. This may reflect unique geographical or dietary exposure backgrounds in our cohort, which should be validated in independent populations.

### Analysis of thyroid function indicators

Following the assessment of blood trace elements, the thyroid function profile was evaluated to offer a comprehensive metabolic overview. The results, as detailed in **Table 4**, reveal a complex and predominantly abnormal thyroid status among the subset of the study population who underwent this test. A remarkably high prevalence of values beyond the reference ranges was noted for several key indicators. Notably, all samples of both Total Triiodothyronine (TT3) and Total Thyroxine (TT4) were abnormal but deviated in opposite directions (100% exceeded standard for TT3, primarily below standard for TT4). This paradoxical combination suggests potential issues with the measurement assay rather than true clinical pathology. In terms of free hormones and TSH, Free Thyroxine (FT4) was below the standard in the majority of cases (59.82%), and TSH was elevated in 34.21% of samples, indicating a high incidence of subclinical or clinical hypothyroidism. Conversely, thyroid autoimmunity was less prevalent, with Thyroperoxidase Antibodies (TPOAb) and Thyroglobulin Antibodies (TGAb) exceeding the standards in only 14.13% and 5.93% of samples, respectively. To further elucidate the interplay between trace elements and thyroid function, we conducted linear regression analyses (presented in the right panels of **Table 3**). These analyses, adjusted for confounding factors, demonstrated significant associations between specific element concentrations and thyroid parameters. For instance, certain elements showed positive standardized β coefficients for TSH and antibody levels (e.g., TPOAb β = 0.276, 95% CI: 0.210–0.342), suggesting that higher environmental exposure to these elements may contribute to thyroid dysfunction independent of overt autoimmune response. Collectively, the data indicate a significant disruption in thyroid hormone homeostasis within the cohort, characterized not only by apparent hypothyroidism but also by quantifiable links to elemental exposure profiles.

**Table 3:** Statistical analysis of plasma element concentrations in thyroid disease patients (case) and healthy controls (control).

Element	Control mean (μg/L)	Case mean (μg/L)	Overall mean (μg/L)	Overall SD (μg/L)	Difference (%)
Vanadium(V)	0.2336	0.2761	0.24	0.631	18.19
Chromium (Cr)	1.91	2.28	2.24	1.39	19.2
Manganese (Mn)	12.7	14.24	14.08	5.43	12.2
Cobalt (Co)	0.25	0.24	0.24	0.25	-5.6
Nickel (Ni)	0.63	0.61	0.61	0.66	-3.5
Copper (Cu)	860.03	813.07	817.99	171.45	-5.5
Zinc (Zn)	5.74	6.40	6.33	1.74	11.6
Gallium (Ga)	0.22	0.17	0.18	0.22	-22.2
Arsenic (As)	2.05	2.27	2.25	1.60	11.0
Selenium (Se)	153.78	159.84	159.21	114.70	3.9
Strontium (Sr)	24.63	24.64	24.64	8.01	0
Cadmium (Cd)	2.06	1.13	1.23	1.37	-45.0
Tin (Sn)	0.00	0.05	0.05	0.17	1094.9
Antimony (Sb)	0.03	0.03	0.03	0.10	-3.5
Barium (Ba)	56.23	50.57	51.15	25.94	-10.1
Mercury (Hg)	0.00	3.58	3.21	23.67	78569.7
Thallium (Tl)	0.05	0.07	0.07	0.07	29.1
Plumbum (Pb)	19.07	15.09	15.51	11.87	-20.8

derived from independent samples t-tests.

**Table 4:** Measurement results and statistical analysis of thyroid function indicators in the study population. .

Indicator	Standard	• Total sample	Values below the standard	Values above the standard	Quantity exceeds the standard	Percentage exceeding the standard (%)	Mean value	Standardized β Coefficient	95% CI for β​
TSH	0.4 - 4.0 mIU/L	760	32	228	260	34.21	3.46 mIU/L	0.152​	0.089 - 0.215​
TT3	0.8 - 2.0 nmol/L	760	0	760	760	100	98.4 nmol/L	-0.087​	-0.142 - -0.032​
TT4	60–160 nmol/L	760	758	2	760	100	22.87 nmol/L	-0.231​	-0.298 - -0.164​
FT3	3.5 - 6.5 pmol/L	760	51	50	101	13.29	5.1 pmol/L	0.045​	-0.011 - 0.101​
FT4	11.5 - 22.5 pmol/L	759	437	17	454	59.82	35.04 pmol/L	0.198​	0.131 - 0.265​
TPOAb	0–35 IU/mL	757	0	107	107	14.13	48.1 IU/mL	0.276​	0.210 - 0.342​
TRAb	0 - 1.75 IU/L	25	0	3	3	12	4.26 IU/L	0.189​	-0.012 - 0.390​
TGAb	0–115 IU/mL	759	0	45	45	5.93	51 IU/mL	0.312​	0.245 - 0.379​

### Association of BRAF V600E mutation with alterations in trace element profiles

Analysis of major driver gene mutations, including TERT promoter (C228T, C250T), KRAS, and NRAS, identified BRAF V600E as the most frequently altered event, occurring in 410 (51.1%) of 801 tested cases. The association between BRAF V600E mutation status and the concentrations of 18 trace elements is detailed in **Table 5**. Comparative analysis revealed that none of the elements exhibited a statistically significant difference (P < 0.05) between the mutated and non-mutated groups. However, several non-significant trends were observed. The most notable differences were seen in Cadmium and Mercury, where the mean concentration was 12% (ratio=1.12) and 22% (ratio=1.22) higher in the BRAF-mutated group, respectively, though with P-values of 0.19 and 0.28. A similar pattern of elevated levels in the mutated group was observed for Strontium, Barium, and Plumbum (all with a mean ratio of 1.05-1.06). Conversely, elements such as Stannum and Thallium showed substantially lower mean concentrations in the mutated group (ratios of 0.74 and 0.81, respectively). Overall, these findings suggest that while the BRAF V600E mutation is not strongly associated with significant alterations in the systemic levels of most trace elements, there may be a potential, non-significant trend towards higher bodily burden of certain non-essential elements like Cadmium and Mercury in BRAF-mutated individuals, warranting further investigation in larger cohorts.

**Table 5:** Comparison of trace element concentrations (μg/L) between subjects with the BRAF V600E mutation and those with the wild-type gene.

Element	Mean value of mutated (μg/L)	Mean value of non-mutated (μg/L)	Mean difference	Mean ratio (%)	P value
Vanadium	0.22	0.25	-0.03	0.87	0.50
Chromium	2.45	2.35	0.11	1.05	0.34
Manganese	12.93	12.75	0.17	1.01	0.60
Cobalt	0.18	0.16	0.01	1.09	0.39
Nickel	0.79	0.89	-0.10	0.89	0.16
Cuprum	757.57	772.14	-14.57	0.98	0.30
Zinc	9.03	8.64	0.39	1.04	0.35
Gallium	0.25	0.26	-0.01	0.94	0.56
Arsenic	2.19	2.28	-0.09	0.96	0.71
Selenium	216.30	211.04	5.25	1.02	0.34
Strontium	31.20	29.74	1.46	1.05	0.13
Cadmium	1.14	1.01	0.12	1.12	0.19
Stannum	0.36	0.49	-0.12	0.74	0.18
Stibium	0.19	0.22	-0.03	0.89	0.35
Barium	62.16	58.94	3.22	1.05	0.20
Mercury	3.82	3.14	0.68	1.22	0.28
Thallium	0.07	0.08	-0.01	0.81	0.15
Plumbum	14.14	13.35	0.78	1.06	0.18

The mean difference is calculated as (Mean value of mutated - Mean value of non-mutated). The mean ratio is calculated as (Mean value of mutated/Mean value of non-mutated). P-values were derived from independent samples t-tests.

### Association of mercury body burden with mutation status in the cohort

**Figure 4:**
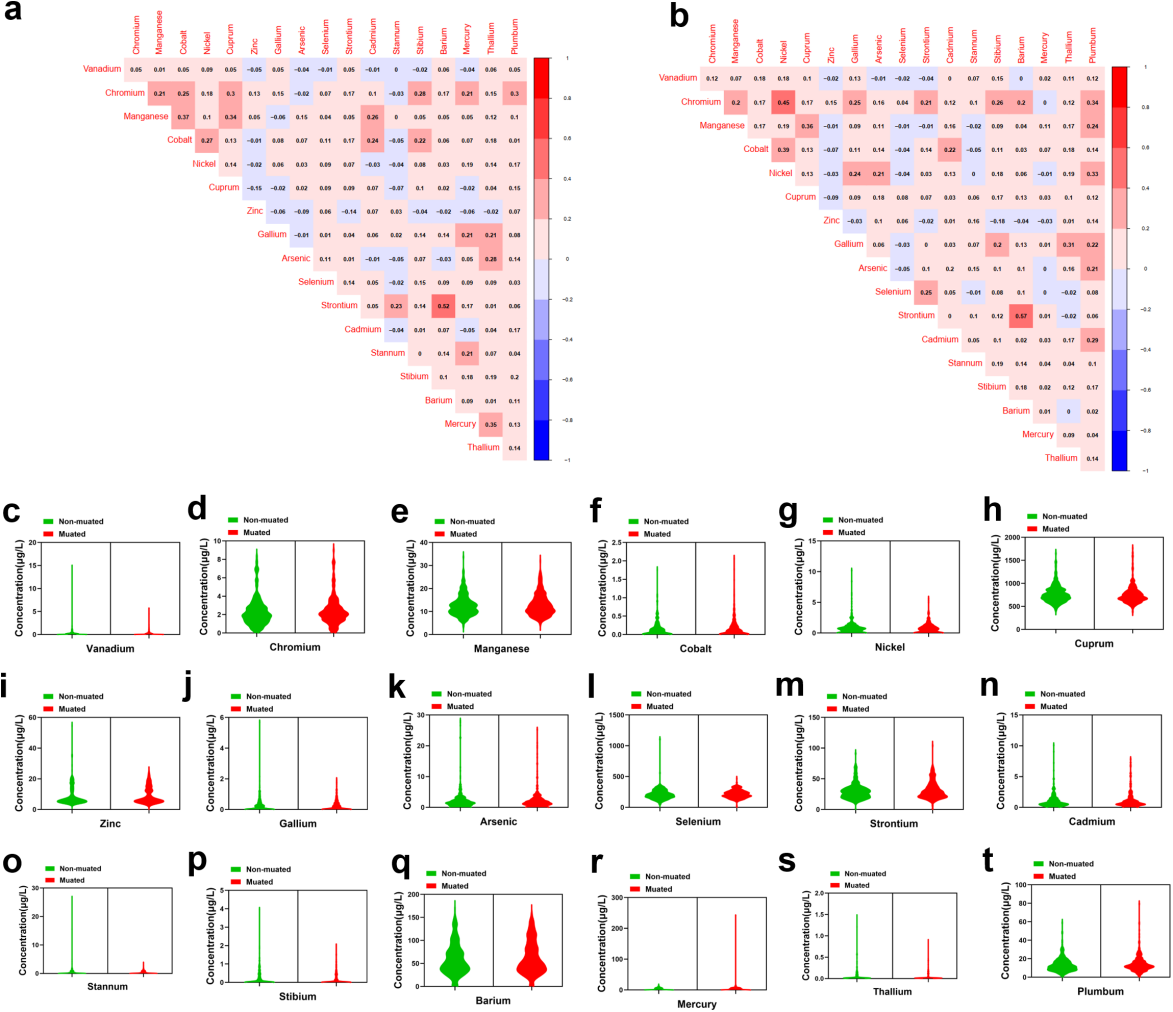
Comparative analysis of trace metal levels relative to BRAF V600E mutation status. Subgroup heatmaps comparing the elemental profiles between the non-mutated **(A)** and mutated **(B)** groups, visualized using the same color scaling as in **(C–T)** violin plots illustrating the distribution and concentration of each individual trace element (vanadium, chromium, manganese, vobalt, nickel, cuprum, zinc, gallium, arsenic, selenium, strontium, cadmium, stannum, stibium, barium, mercury, thallium, plumbum) stratified by BRAF V600E mutation status. For each element, the left plot (green) corresponds to the non-mutated group, and the right plot (red) corresponds to the mutated group, allowing for visual comparison of central tendency and data spread between the two cohorts.

**Figure 4** presents a comparative analysis of trace elemental profiles stratified by BRAF V600E mutation status. The pairwise correlation heatmaps reveal that the overall architecture of inter-element associations is largely conserved between the non-mutated (**Figure 4A**) and mutated (**Figure 4B**) subgroups. However, a specific and notable alteration is observed in the correlation between Barium (Ba) and Strontium (Sr). In the non-mutated group, the Ba-Sr correlation coefficient is 0.52, a value consistent with the general pattern previously observed in the broader thyroid cancer cohort. In contrast, within the BRAF V600E mutated subgroup, this correlation strengthens to 0.57. This elevated association, compared to the 0.55 observed in the unstratified patient population, suggests a potential specific link between the coordinated behavior of these two elements and the presence of the BRAF V600E mutation. Concurrently, analysis of individual element concentrations (**Figures 4C–T**) identified significant alterations associated with the mutation. Our analysis revealed distinct alterations in trace element concentrations associated with BRAF V600E mutation status. Notably, the initial statistical outcomes were influenced by a limited number of outlier values. In the Hg analysis, a single extreme value in the mutant group (241.39 μg/L) strongly influenced the comparison. Retrospective dietary analysis of these outliers indicated that the subject with extraordinarily elevated Hg levels consistently consumed two dietary sources that had been previously associated with mercury exposure: abyssal fish (e.g., jellyfish) and Youtiao, which were the primary contributing factors to the observed high mercury concentration. The preserved global correlation pattern suggests that fundamental homeostatic networks of trace elements remain largely intact regardless of BRAF status. However, the specific enhancement of the Ba-Sr correlation in the mutated subgroup may indicate a mutation-influenced co-regulation or shared exposure pathway. The significant decrease in Sn levels could imply an altered utilization or depletion of this element in the context of the mutated oncogenic pathway. The marked accumulation of Hg in the mutated group is particularly noteworthy, potentially reflecting a differential toxic metal retention or a mutation-associated disruption in detoxification processes. These distinct elemental signatures—the modulated Ba-Sr relationship, Sn depletion, and Hg accumulation—collectively point to a unique biochemical environment associated with BRAF V600E mutant thyroid tumors, which may interplay with disease progression or therapeutic response. Further investigation is required to elucidate the underlying mechanisms and clinical implications of these observations.

## Discussion

This study provides a comprehensive analysis of the associations between plasma mineral element profiles and the risk of thyroid tumors, integrating insights from dietary habits and molecular characteristics such as the BRAF V600E mutation. Our findings reveal pronounced differences in elemental concentrations between patients with thyroid diseases and healthy controls, highlighting the potential influence of environmental exposures on thyroid tumorigenesis. These results are consistent with previous research that highlights the importance of trace elements in endocrine disorders. Moreover, we introduce novel aspects, specifically the interaction between dietary factors and elemental imbalances, which necessitates further elaboration. By comparing our data with the data regarding metallic elements and thyroid tumors, and taking into account the genomic analysis of thyroid cancer that associates metal accumulation with tumor heterogeneity, we can place our observations within broader environmental and molecular frameworks (42, 43). We will concentrate on interpreting the key results, emphasizing their implications, and delving into potential mechanisms, all the while addressing the study’s strengths and limitations.

First, the significantly altered plasma elemental profiles in thyroid cancer patients, characterized by lower levels of essential elements like selenium and manganese and higher levels of non-essential elements such as barium, suggest a disruption in homeostatic balance that may contribute to pathogenesis. These findings are consistent with the currently reported lower urinary concentrations of chromium, manganese, nickel, and selenium in thyroid tumor cases compared to controls, suggesting that systemic elemental deficiencies may be potentially associated with an increased risk of the disease (42). The consistency observed across different biofluids, specifically blood plasma and urine (42), further strengthens the validity of metallic elements as biomarkers for thyroid disorders. Notably, the heatmaps of **Figures 2b**, **4a, b** revealed a conserved network of element interactions. However, there were alterations, such as the strengthened barium-strontium correlation in patients, which implies that disease-specific metabolic changes may amplify certain exposure pathways, suggesting that elemental imbalances could serve as early indicators of cellular stress across cancer types (43). The lower levels of essential elements in cohort may reflect an increased utilization or excretion resulting from oxidative stress mechanisms. This situation warrants an investigation into antioxidant supplementation as a preventive strategy.

Second, the profound influence of dietary habits on elemental concentrations stands out as a crucial factor. Certain specific foods, like Youtiao (fried dough), are linked to a 153% increase in mercury levels and are also associated with elevated plumbum and arsenic levels. This finding supports the idea that lifestyle factors can substantially change the body burdens of toxic elements. Our stratification based on diet indicated that preferences for salty, greasy, or preserved foods resulted in heterogeneous elemental distributions, which might interact with genetic susceptibilities to regulate disease risk. For instance, the elevated mercury levels associated with Youtiao consumption may stem from contamination during the frying process, which is consistent with the emphasis on environmental sources such as seafood that contribute to metal accumulation. These findings highlight the necessity for public health interventions aimed at dietary sources of toxic elements, along with personalized nutrition advice for high - risk populations, to alleviate the burden of thyroid disease. Although rigorous quality control was implemented, the absolute concentrations of certain elements (e.g., manganese) should be validated in future studies with similar populations and analytical methodologies.

Thirdly, the high prevalence of abnormal thyroid function parameters, such as elevated TSH and decreased FT4, suggests significant endocrine disruption in our cohort, which might be partially attributed to elemental exposures. The inverse relationship between TT3 and TT4 levels indicates complex hormonal dysregulation, potentially due to element-induced interference in hormone synthesis or secretion. In our study, the non-significant tendency of higher mercury and cadmium levels in BRAF-mutated cases parallels the finding that mercury concentration is correlated with an increased mutation burden in colorectal cancer, which implies that toxic metallic elements may promote genomic instability. Although our results did not achieve statistical significance, potentially because of the sample size, the observed trend is consistent with mechanistic studies suggesting that metallic elements such as cadmium can cause DNA damage and disrupt repair pathways, thus promoting mutations in oncogenes like BRAF. This interaction between elemental exposure and molecular pathogenesis calls for further investigation in larger cohorts to elucidate causal relationships.

The stronger barium-strontium correlation and lower tin levels in mutated subgroups suggest that specific elemental signatures may be associated with distinct genetic alterations. The accumulation of mercury in BRAF-mutated patients may indicate a selective pressure or detoxification deficiency. These parallels underscore the potential of integrating environmental biomarkers with genomic data to enhance risk stratification and therapeutic strategies, for instance, by targeting metal detoxification pathways in mutation-driven subtypes.

The broader implications of our findings relate to the mechanistic pathways through which metallic elements influence thyroid tumorigenesis, such as oxidative stress, immune modulation, and endocrine disruption. The conserved inter-element correlations observed in **Figure 2** imply the existence of underlying homeostatic networks, whereas the disease - specific shifts indicate pathogenic disruptions. Our results expand on this by establishing a connection between dietary habits and these pathways, providing a comprehensive perspective on exposure-disease interactions. However, limitations encompass the design and the emphasis on blood plasma elements, which might not comprehensively capture tissue-level exposures. Future studies ought to integrate longitudinal data, multi-omics approaches, and experimental models to validate these associations and explore therapeutic targets, for example, selenium supplementation to mitigate mercury toxicity.

## Conclusion

In summary, this study comprehensively elucidates the intricate interplay among mineral element exposures, dietary habits, and molecular features in thyroid tumors. It reveals distinct elemental profiles and how they are modified by lifestyle factors. The associations with BRAF mutations and thyroid dysfunction offer novel insights into the environmental determinants of tumor heterogeneity, which is consistent with broader cancer research trends. Key aspects include the identification of diet as a major source of exposure, the potential for elemental signatures to mirror genetic alterations, and the emphasis on integrated approaches for prevention. We situate our findings within the contexts of environmental health and genomics, highlighting the translational potential of element - based biomarkers. Future research should prioritize prospective cohort studies and mechanistic investigations to confirm these relationships and develop targeted interventions, thereby ultimately reducing the global burden of thyroid cancer.

## Data Availability

The datasets presented in this article are not readily available because the datasets generated and/or analyzed during the current study are not publicly available but are available from the corresponding author on reasonable request. Requests to access the datasets should be directed to Chao Zhai, sxzhaichao@163.com.
